# Anethole improves the developmental competence of porcine embryos by reducing oxidative stress via the sonic hedgehog signaling pathway

**DOI:** 10.1186/s40104-022-00824-x

**Published:** 2023-02-22

**Authors:** Ye Eun Joo, Pil-Soo Jeong, Sanghoon Lee, Se-Been Jeon, Min-Ah Gwon, Min Ju Kim, Hyo-Gu Kang, Bong-Seok Song, Sun-Uk Kim, Seong-Keun Cho, Bo-Woong Sim

**Affiliations:** 1 Futuristic Animal Resource and Research Center, Korea Research Institute of Bioscience and Biotechnology, Cheongju, South Korea; 2 Department of Animal Science, College of Natural Resources and Life Science, Pusan National University, Miryang, South Korea; 3 Laboratory of Theriogenology, College of Veterinary Medicine, Chungnam National University, Daejeon, South Korea; 4 Department of Biotechnology, College of Engineering, Daegu University, Gyeongsan, South Korea; 5 Department of Animal Science and Biotechnology, College of Agriculture and Life Science, Chungnam National University, Daejeon, South Korea; 6 Department of Functional Genomics, University of Science and Technology, Daejeon, South Korea; 7 Department of Animal Science, College of Natural Resources and Life Science, Life and Industry Convergence Research Institute, Pusan National University, Miryang, South Korea

**Keywords:** Anethole, Lipid metabolism, Mitochondrial function, Porcine embryo development, Sonic hedgehog signaling pathway

## Abstract

**Background:**

Anethole (AN) is an organic antioxidant compound with a benzene ring and is expected to have a positive impact on early embryogenesis in mammals. However, no study has examined the effect of AN on porcine embryonic development. Therefore, we investigated the effect of AN on the development of porcine embryos and the underlying mechanism.

**Results:**

We cultured porcine in vitro-fertilized embryos in medium with AN (0, 0.3, 0.5, and 1 mg/mL) for 6 d. AN at 0.5 mg/mL significantly increased the blastocyst formation rate, trophectoderm cell number, and cellular survival rate compared to the control. AN-supplemented embryos exhibited significantly lower reactive oxygen species levels and higher glutathione levels than the control. Moreover, AN significantly improved the quantity of mitochondria and mitochondrial membrane potential, and increased the lipid droplet, fatty acid, and ATP levels. Interestingly, the levels of proteins and genes related to the sonic hedgehog (SHH) signaling pathway were significantly increased by AN.

**Conclusions:**

These results revealed that AN improved the developmental competence of porcine preimplantation embryos by activating SHH signaling against oxidative stress and could be used for large-scale production of high-quality porcine embryos.

**Supplementary Information:**

The online version contains supplementary material available at 10.1186/s40104-022-00824-x.

## Background

Infertility is a global health problem-48 million couples and 186 million individuals currently suffer from infertility [1]. The treatment modalities for infertility are drugs, surgical procedures, and assisted reproductive technologies (ARTs). ARTs encompasses intrauterine insemination and in vitro fertilization (IVF), which has the highest success rate [2, 3]. For successful ARTs, morphologic parameters linked to the viability of pronuclear, cleavage stage, and blastocyst stage preimplantation embryos are used to evaluate embryo quality [4]. However, the use of morphologic parameters is not widespread because of significant differences between the in vivo microenvironment and in vitro culture (IVC) systems, including increased oxidative stress in the latter [5]. Therefore, use of optimized embryo culture conditions is important to maximize the success rate of ARTs.

Reactive oxygen species (ROS), created by intracellular energy metabolism, are associated with embryonic development. Excessive ROS causes oxidative stress, leading to DNA damage, mitochondrial dysfunction, lipid peroxidation, adenosine 5′-triphosphate (ATP) depletion, apoptosis, and embryonic developmental arrest [6, 7]. In vivo, the female reproductive tract prevents ROS formation and ROS damage by means of antioxidant systems [8]. IVC systems require the addition of antioxidants to the medium to reduce oxidative stress. Supplementation with exogenous antioxidants such as lycopene [8], laminarin [9], vitamin C [10], and imperatorin [11] could improve the quality of preimplantation porcine embryos. However, current IVC systems do not fully recapitulate the in vivo environment. Therefore, it is necessary to clarify the mechanisms of antioxidant activity and develop more effective antioxidants.

Anethole (AN), also known as anise camphor, is the major component of the essential oil extracted from the plant *Croton zehntneri* (Euphorbiaceae) and is used as a flavoring agent in the food industry (e.g., alcoholic drinks, baked goods, ice-creams, and cakes) [12]. AN is also used in the cosmetics and pharmaceutical industries as a result of its anti-inflammatory, anesthetic, anticarcinogenic, antibacterial, and estrogenic activities [13, 14]. It has been shown experimentally to be safe at low doses because it is non-toxic, non-genotoxic, and non-carcinogenic [15]. AN acts as an antioxidant in various human cancer cell lines and human mesenchymal stem cell lines [16–18], and in the female reproductive system [19–22]. In goat, AN supplementation during IVC of preantral follicles increased follicular development and oocyte meiotic maturation by reducing the ROS level [19, 20]. In addition, AN supplementation during oocyte maturation improved bovine mitochondrial function and embryonic development [21]. Moreover, AN increases the activity of the primary antioxidants superoxide dismutase (SOD), catalase (CAT), and glutathione peroxidase (GPX), as well as cellular glutathione (GSH) [23]. Although AN supplementation exerts beneficial effects in the female reproductive system of several species, its roles in porcine early embryonic development and the related signaling pathways are unclear.

The sonic hedgehog (SHH) signaling pathway is the major mediator of embryonic development [24], and enhances cell proliferation and differentiation in tissues and organs, including reproductive tissue, via paracrine signaling [25]. In vertebrates, the hedgehog signaling pathway has three components-SHH, Indian hedgehog, and Desert hedgehog [26]. The SHH signaling pathway is activated by the interaction of two surface membrane proteins-patched (PTCH) and smoothened (SMO)-which activate the downstream signaling gene, glioma-associated oncogene homolog (GLI) [25, 27]. During development, the inhibition of SMO is relieved by binding the SHH ligand to PTCH receptor on the cell surface. Consequently, activated SMO triggers transcription of GLI, leading to upregulation of genes (*PTCH*, *SMO*, and *GLI*) that regulate cell patterning, proliferation, migration, and differentiation [28]. Genes and proteins related to the SHH signaling pathway are expressed in porcine parthenogenetic embryos at different developmental stages [25, 29]. In addition, SHH promoted the preimplantation development of IVF porcine embryos [29].

We investigated the role of AN in embryonic development in pigs based on their anatomical, physiological, and genetic similarities with human [30]. The objective was to examine the effect of AN supplementation on porcine embryonic development by evaluating developmental competence, intracellular ROS and GSH levels, mitochondrial function, and lipid metabolism. In addition, we examined the SHH signaling pathway to investigate the mechanism by which AN influences porcine early embryogenesis.

## Methods

### Chemicals

All chemicals and reagents used in this study were purchased from Sigma-Aldrich Chemical Company (St. Louis, MO, USA), unless otherwise stated.

### Oocyte collection and in vitro maturation (IVM)

Porcine ovaries were collected from a local abattoir and transported to the laboratory in 0.9% saline containing 75 μg/mL benzyl-penicillin potassium and 50 μg/mL streptomycin sulfate salt at 38.5 °C. Cumulus oocyte complexes (COCs) were aspirated from 3 to 6 mm follicles using a disposable 10-mL syringe with an 18-gauge needle. COCs were washed three times in 0.9% saline containing 1 mg/mL bovine serum albumin (BSA) and 50 COCs were cultured in IVM medium (Tissue Culture Medium 199 with 10% porcine follicular fluid, 10 IU/mL pregnant mare serum gonadotropin, 10 IU/mL human chorionic gonadotropin, 0.57 mmol/L cysteine, 25 μmol/L β-mercaptoethanol, and 10 ng/mL epidermal growth factor) for 44 h at 38.5 °C in an atmosphere of 5% CO_2_. After 22 h of maturation, COCs were cultured in IVM medium lacking hormones for another 22 h. After 44 h of IVM, expanded cumulus cells were removed by gently pipetting with 0.1% hyaluronidase. Metaphase II oocytes with a visible polar body, regular morphology, and homogenous cytoplasm were used for experiments.

### IVF and IVC

IVF medium consisted of modified Tris-buffered medium containing 113.1 mmol/L NaCl, 3 mmol/L KCl, 7.5 mmol/L CaCl_2_·2H_2_O, 20 mmol/L Tris (Fisher Scientific, Waltham, MA, USA), 11 mmol/L glucose, 5 mmol/L sodium pyruvate, 2.5 mmol/L caffeine sodium benzoate, and 1 mg/mL BSA. Oocytes (10–15) were transferred to 48-μL droplets of IVF medium under paraffin oil. Next, fresh porcine spermatozoa were washed three times with Dulbecco’s phosphate-buffered saline (DPBS; Gibco, Grand Island, NY, USA) containing 100 μg/mL benzyl-penicillin potassium, 75 μg/mL streptomycin sulfate, and 1 mg/mL BSA. The spermatozoa were resuspended in IVF medium to a final concentration of 1.5 × 10^5^/mL. Next, 2 μL of diluted spermatozoa was added to IVF medium, and co-incubated with oocytes for 6 h at 38.5 °C in an atmosphere of 5% CO_2_. After 6 h, spermatozoa attached to oocytes were stripped by successive pipetting. Next, IVF embryos were transferred to 40-μL droplets of IVC medium, which consisted of porcine zygote medium-3 with 4 mg/mL BSA, at 38.5 °C in an atmosphere of 5% CO_2_. The cleavage and blastocyst formation rates were evaluated at 48 and 144 h after culture, respectively.

### Chemical treatment

To evaluate the effect of AN on porcine embryonic development, IVF embryos were cultured in IVC medium with AN (0, 0.3, 0.5, and 1 mg/mL) at 38.5 °C in an atmosphere of 5% CO_2_ for 6 d. We first conducted preliminary experiments to determine range of AN concentrations, based on previous studies [20–22]. We chose 0.5 mg/mL as the optimal concentration of AN after results of various concentrations for the blastocyst formation rate, and we used 0.5 mg/mL AN for the following experiments. To further confirm that effects of AN on SHH signaling pathway, the concentrations of cyclopamine (2 μmol/L) was set according to previous study [25].

### Terminal deoxynucleotidyl transferase-mediated dUTP digoxygenin nick-end labeling assay

To detect apoptotic cells, blastocysts were stained using an In Situ Cell Death Detection Kit (Roche, Basel, Switzerland). The blastocysts were fixed in formalin solution overnight at 4 °C and permeabilized by incubation with 1% Triton X-100 in DPBS at room temperature (RT) for 1 h. Next, the blastocysts were washed three times in DPBS supplemented with 1 mg/mL polyvinyl alcohol (PVA) (DPBS/PVA) and incubated with fluorescein-conjugated dUTP and terminal deoxynucleotidyl transferase in the dark at 38.5 °C for 1 h. Subsequently, the blastocysts were washed three times with DPBS/PVA and mounted on clean slide glasses with 4,6-diamidino-2-phenylindole (DAPI; Vector Laboratories Inc., Burlingame, CA, USA). The total number of nuclei and the numbers of apoptotic nuclei were observed using a fluorescence microscope (DMi8; Leica, Wetzlar, Germany).

### CDX2 staining

Blastocysts were fixed in formalin solution overnight and washed three times in DPBS/PVA. Fixed blastocysts were incubated for 1 h in DPBS containing 1% Triton X-100 at RT and washed in DPBS/PVA. Next, blastocysts were stored in DPBS/PVA containing 1 mg/mL BSA (blocking solution) at 4 °C for 6 h and blocked in DPBS supplemented with 10% normal goat serum for 1 h at RT. The blastocysts were incubated at 4 °C overnight with undiluted mouse monoclonal CDX2 primary antibody (BioGenex Laboratories Inc., San Ramon, CA, USA). The blastocysts were washed three times in blocking solution and incubated at RT for 1 h with the secondary Alexa-Fluor-488-labeled goat anti-mouse IgG antibody (1:200). Subsequently, the blastocysts were washed three times in blocking solution and mounted on clean slide glasses with DAPI. DAPI-labeled or CDX2-positive cells were observed using a fluorescence microscope (DMi8; Leica).

### Measurement of intracellular ROS and GSH levels

Intracellular ROS and GSH levels were measured with CM-H2DCFDA (Invitrogen, Carlsbad, CA, USA) and CMF2HC (Invitrogen), respectively. D2 embryos and D6 blastocysts were incubated for 30 min in DPBS/PVA containing 5 μmol/L CM-H2DCFDA or 10 μmol/L CMF2HC. After incubation, embryos and blastocysts were washed in DPBS/PVA, and fluorescence was observed using a fluorescence microscope (DMI 4000B; Leica) with ultraviolet filters (460 nm for ROS and 370 nm for GSH). The fluorescence signal intensities were analyzed using ImageJ software (version 1.47; National Institutes of Health, Bethesda, MD, USA) after normalization through subtraction of the background intensity to that of control embryos.

### Quantitative real-time polymerase chain reaction (qPCR)

Poly(A) mRNAs were extracted from 20 D2 embryos and 10 D6 blastocysts using a Dynabeads mRNA Direct Kit (Invitrogen) according to the manufacturer’s instructions. Samples were lysed in 100 μL of lysis/binding buffer at RT for 5 min, and 30 μL of Dynabeads oligo (dT)25 was used to separate mRNAs. The beads were hybridized for 5 min and separated from the binding buffer using a Dynal magnetic bar (Invitrogen). Bound poly(A) mRNAs and beads were washed in buffers A and B individually, and 7 μL of Tris–HCl buffer was added to separate poly(A) mRNAs. The poly(A) mRNAs were reverse-transcribed using a PrimeScript™ RT Reagent Kit with gDNA Eraser (Takara Bio Inc., Shiga, Japan). Reverse transcription was carried out at 37 °C for 15 min and 85 °C for 5 s. The synthesized cDNA was used as the template for qPCR at 95 °C for 5 min and 95 °C for 20 s and 60 °C for 20 s. For the comparative analyses, mRNA expression levels were normalized to *H2A* and are expressed as the fold change. The sample delta Ct (SΔCT) value was calculated from the difference between the Ct values of *H2A* and the target genes. The relative gene expression levels between the samples and the controls were determined using the formula 2^−(SΔCT−CΔCT)^. All qPCR results were performed three independent experiments with different sets of embryos or blastocysts. The primers used are listed in Additional file 1: Table S1.

### Measurement of mitochondrial distribution and mitochondrial membrane potential

The distribution of active mitochondria and the mitochondrial membrane potential were measured using MitoTracker Red CMXRos (Invitrogen) and tetramethylrhodamine, methyl ester TMRM (Invitrogen). D2 embryos and D6 blastocysts were incubated in DPBS/PVA containing 200 nmol/L MitoTracker or 200 nmol/L TMRM at 38.5 °C for 30 min and washed three times with DPBS/PVA. The embryos and blastocysts were observed using a fluorescence microscope (DMi8; Leica) for MitoTracker staining or were incubated with 10 μg/mL Hoechst 33342 at 38.5 °C for 10 min and observed using a fluorescence microscope for TMRM staining. The fluorescence signal intensities were analyzed using ImageJ software after normalization through subtraction of the background intensity to that of control embryos.

### Lipid droplet, fatty acid, and ATP staining

D2 embryos were fixed in formalin solution overnight at 4 °C. Fixed embryos were washed three times in DPBS/PVA and treated with 10 μg/mL BODIPY 493/503, 6 μmol/L BODIPY 558/568C12, or 0.5 μmol/L BODIPY FL ATP for 1 h at RT. Stained embryos were washed three times in DPBS/PVA and incubated with 10 μg/mL Hoechst 33342 for 10 min at 38.5 °C. The embryos were observed using a fluorescence microscope. The fluorescence signal intensities were analyzed using ImageJ software after normalization through subtraction of the background intensity to that of control embryos.

### Immunocytochemical staining

To stain SHH signaling proteins, D2 embryos were fixed in formalin solution overnight and washed three times in DPBS/PVA. Fixed embryos were incubated at RT for 1 h in DPBS containing 1% Triton X-100. Subsequently, embryos were washed three times in DPBS/PVA and stored in DPBS/PVA containing 1 mg/mL BSA (blocking solution) at RT for 1 h. The embryos were incubated with primary antibodies for SHH (1:100; sc-365112; Santa Cruz Biotechnology, Inc., Santa Cruz, CA, USA), SMO (1:100; sc-13943; Santa Cruz), PTCH1 (1:200; sc-9016; Santa Cruz), or GLI1 (1:200; sc-20687; Santa Cruz) at 4 °C overnight. After washing three times in DPBS/PVA, embryos were placed in blocking solution for 1 h at RT. Next, the embryos were reacted at RT for 1 h with the Alexa-Fluor-488-labeled goat anti-mouse IgG secondary antibody (200:1). The embryos were washed three times in DPBS/PVA and mounted on clean slide glasses with DAPI. The fluorescence intensities of SHH signaling proteins were observed using a fluorescence microscope (DMi8; Leica). The fluorescence signal intensities were analyzed using ImageJ software after normalization through subtraction of the background intensity to that of control embryos.

### Statistical analysis

Statistical analysis was performed using SigmaStat software (Systat, San Jose, CA, USA). All experiments were repeated at least three times and data are presented as the means ± standard error of the mean. To compare three or more groups, one-way analysis of variance was performed followed by Tukey’s multiple range test. Student’s *t*-test was used to evaluate the significance of between-group differences. Statistical significance was considered at *P* < 0.05.

## Results

### AN improves the developmental competence of porcine IVF embryos

**Fig. 1:**
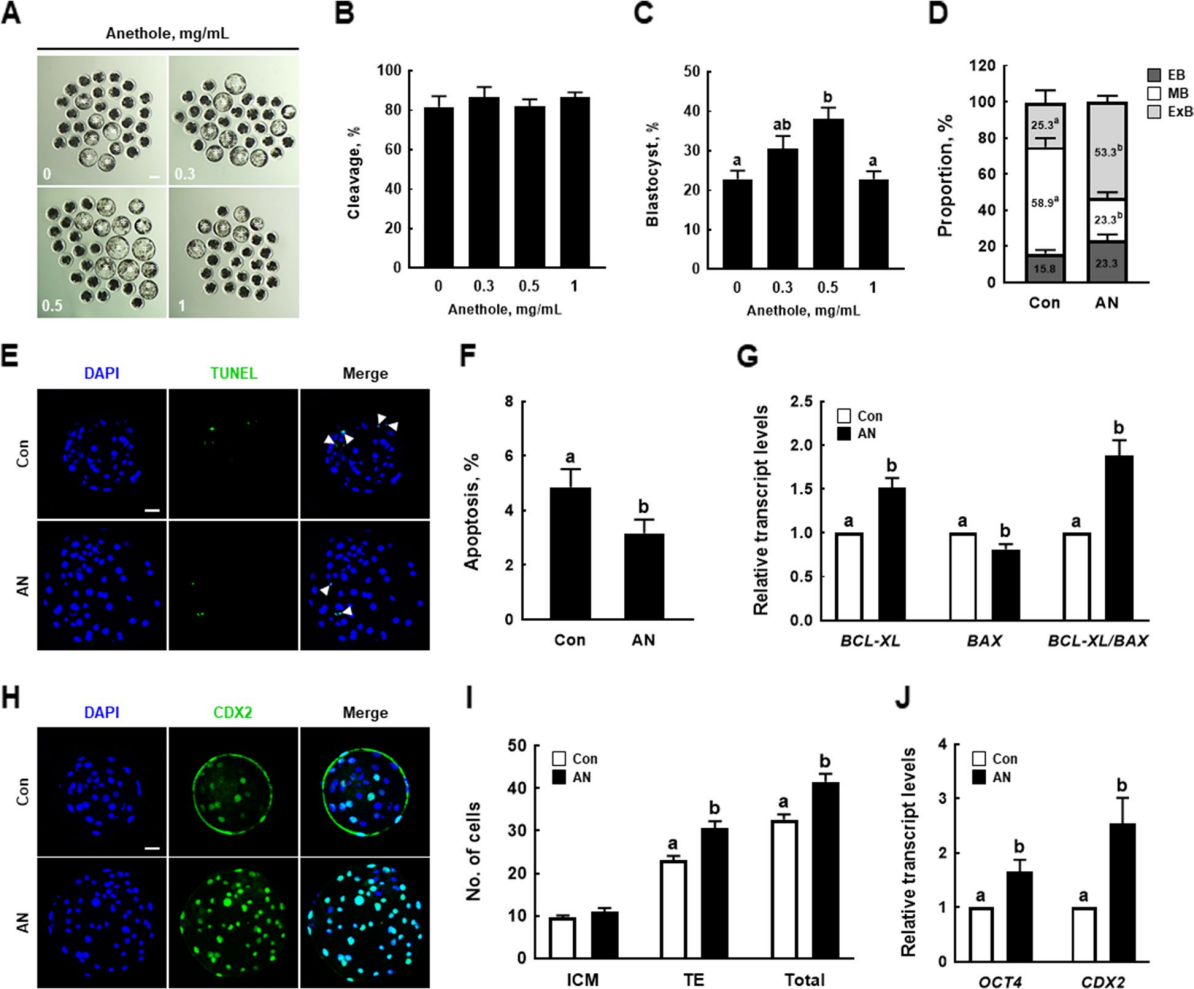
Effect of anethole (AN) on the development of porcine in vitro fertilization (IVF) embryos. **A** Representative images of embryos on D6, **B** percentages of cleavage on D2, and **C** blastocyst formation on D6 (0; *n* = 132, 0.3; *n* = 134, 0.5; *n* = 134, 1; *n* = 133). Scale bar = 100 μm. **D** Proportions of blastocyst stages with and without AN (*n* = 40 per groups). **E** Representative photographs of terminal deoxynucleotidyl transferase-mediated dUTP-digoxygenin staining of blastocysts on D6 and **F** percentages of apoptotic cells with and without AN treatment (*n* = 35 per groups). Scale bar = 50 μm. **G** Relative expression levels of apoptosis-related genes and *BCL-XL*/*BAX* ratio in D6 blastocysts with and without AN treatment (*n* = 3 per groups). **H** Representative images of CDX2 staining of D6 blastocysts and **I** numbers of inner cell masses (ICM), trophectoderms (TE), and total cells in D6 blastocysts with and without AN treatment (*n* = 26 per groups). Scale bar = 50 μm. **J** Relative expression levels of ICM/TE differentiation-related genes in D6 blastocysts with and without AN treatment (*n* = 3 per groups). Data are from five independent experiments, and different superscript letters indicate a significant difference (*P* < 0.05)

We cultured IVF embryos treated with AN (0, 0.3, 0.5, and 1 mg/mL) and assessed the cleavage and blastocyst rates on 2 and 6 d, respectively. There was no difference in the cleavage rate, but the blastocyst formation rate was significantly increased in the 0.5 AN group compared to the control (Fig. 1A–C, Additional file 2: Table S2). The proportion of expanded blastocysts (ExB) was significantly increased in the AN group (Fig. 1D, Additional file 3: Table S3). The proportion of apoptosis was significantly decreased in the AN group compared to the control (Fig. 1E and F, Additional file 4: Table S4). In the AN group, the expression level of *BAX* was significantly reduced, and the expression level of *BCL-XL* and the *BCL-XL*/*BAX* ratio were significantly increased (Fig. 1G). Moreover, there was no significant difference in the number of inner cell mass (ICM) cells, but the numbers of total cells and trophectoderm (TE) cells were significantly increased in the AN group compared to the control (Fig. 1H and I, Additional file 5: Table S5). The expression levels of ICM/TE differentiation-related genes (*OCT4* and *CDX2*) were significantly increased in the AN group (Fig. 1J).

### AN regulates intracellular ROS and GSH levels in porcine IVF embryos

**Fig. 2:**
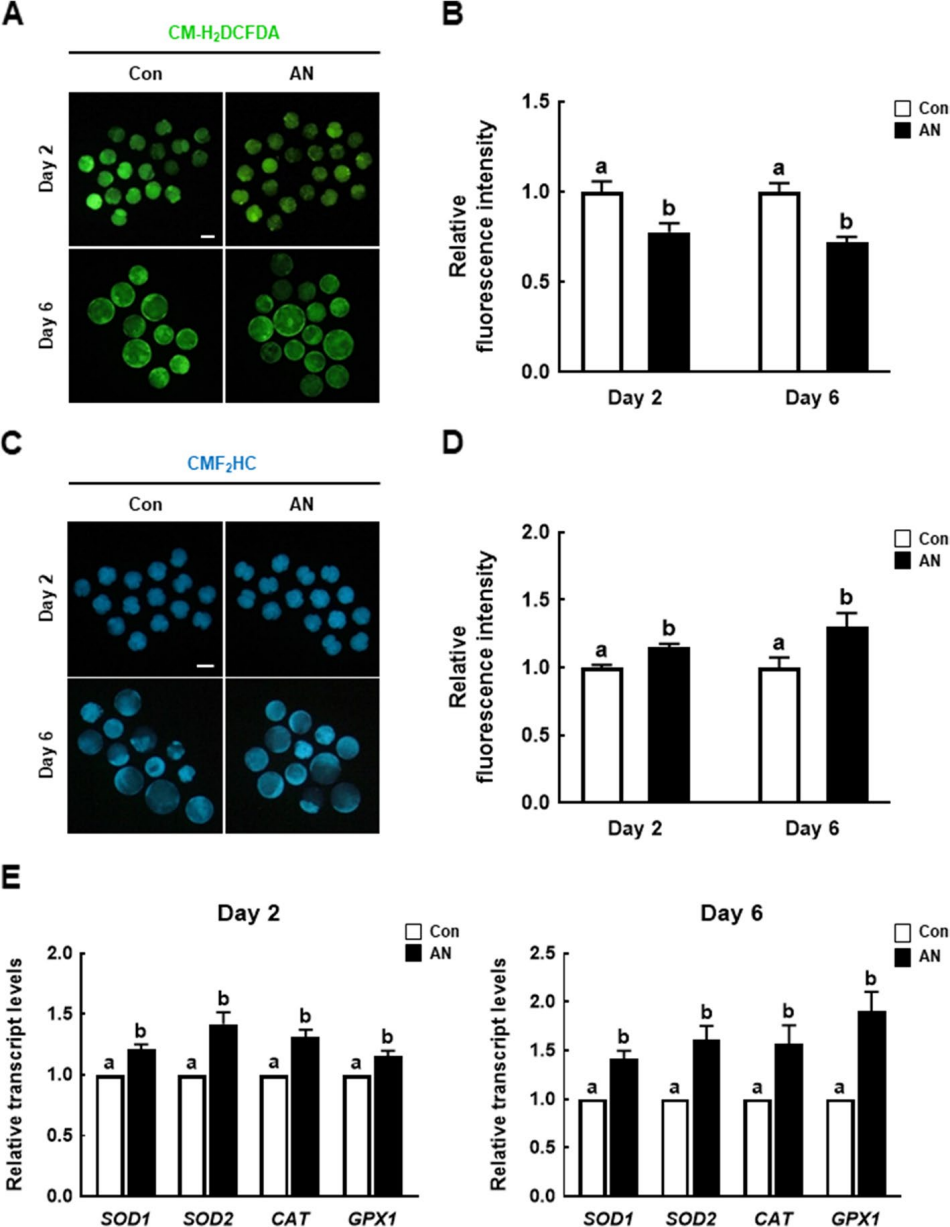
Effect of AN on intracellular reactive oxygen species (ROS) and glutathione (GSH) levels in porcine IVF embryos. **A** Representative fluorescence images of CM-H2DCFDA staining in D2 embryos and D2 blastocysts with and without AN treatment. Scale bar = 100 μm. **B** Relative ROS fluorescence intensity in D2 embryos and D6 blastocysts with and without AN treatment (D2; *n* = 57 per groups, D6; *n* = 34 per groups). **C** Representative fluorescence images of CMF2HC staining of D2 embryos and D6 blastocysts with and without AN treatment. Scale bar = 100 μm. **D** Relative GSH fluorescence intensity in D2 embryos and D6 blastocysts with and without AN treatment (D2; *n* = 44 per groups, D6; *n* = 25 per groups). **E** Relative expression levels of antioxidation-related genes in D2 embryos and D6 blastocysts with and without AN treatment (*n* = 3 per groups). Data are from three independent experiments, and different superscript letters indicate a significant difference (*P* < 0.05)

Next, we investigated the intracellular ROS and GSH levels in D2 embryos and D6 blastocysts. The ROS level was significantly decreased in D2 embryos and D6 blastocysts supplemented with AN compared to the control (Fig. 2A and B), and the GSH level in D2 embryos and D6 blastocysts supplemented with AN was significantly increased compared to the control (Fig. 2C and D). The expression levels of antioxidation-related genes (*SOD1*, *SOD2*, *CAT*, and *GPX1*) were significantly higher in D2 embryos and D6 blastocysts in the AN group (Fig. 2E).

### AN enhances mitochondrial function during porcine IVF embryo development

**Fig. 3:**
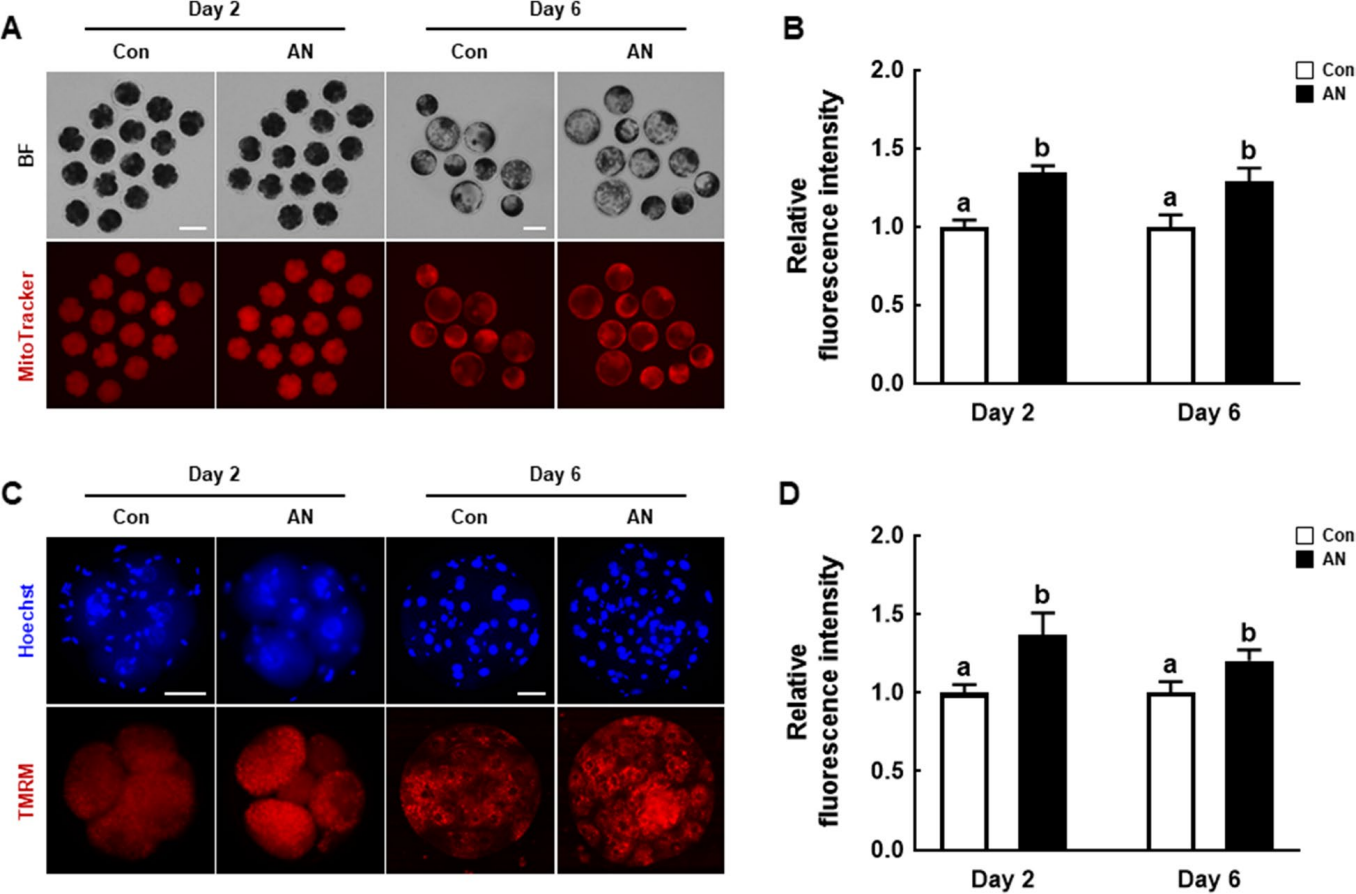
Effect of AN on mitochondrial function in porcine IVF embryos. **A** Representative fluorescence images of MitoTracker Deep Red staining of D2 embryos and D6 blastocysts with and without AN treatment. Scale bar = 100 μm. **B** Relative MitoTracker fluorescence intensity in D2 embryos and D6 blastocysts with and without AN treatment (D2; *n* = 51 per groups, D6; *n* = 25 per groups). **C** Representative TMRM fluorescence images in D2 embryos and D6 blastocysts with and without AN treatment. Scale bar = 50 μm. **D** Relative TMRM fluorescence intensity in D2 embryos and D6 blastocysts with and without AN treatment (D2; *n* = 23 per groups, D6; *n* = 20 per groups). Data are from three independent experiments, and different superscript letters indicate a significant difference (*P* < 0.05)

We examined mitochondrial content and membrane potential in D2 embryos and D6 blastocysts. The MitoTracker intensity was significantly higher in D2 embryos and D6 blastocysts supplemented with AN than in the control (Fig. 3A and B). Consistently, the intensity of TMRM, an index of mitochondrial membrane potential, was significantly higher in D2 embryos and D6 blastocysts treated with AN than in the control (Fig. 3C and D).

### AN regulates lipid metabolism during porcine IVF embryo development

**Fig. 4:**
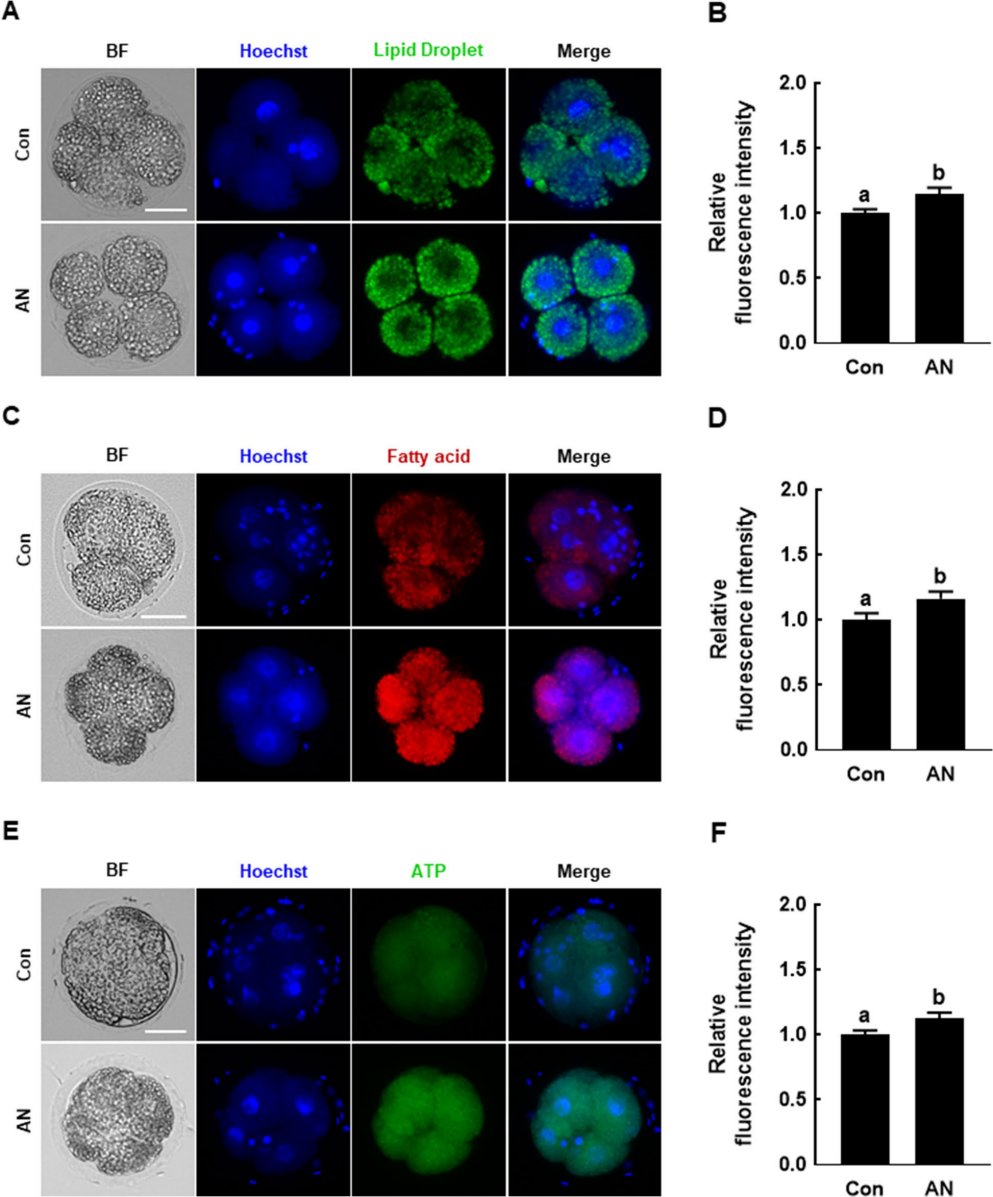
Effect of AN on lipid metabolism in porcine IVF embryos. **A** Representative fluorescence images of lipid droplets in D2 embryos with and without AN treatment. Scale bar = 50 μm. **B** Relative fluorescence intensity of lipid droplets in D2 embryos with and without AN treatment (*n* = 24 per groups). **C** Representative fluorescence images of fatty acids in D2 embryos with and without AN treatment. Scale bar = 50 μm. **D** Relative fluorescence intensity of fatty acid staining in D2 embryos with and without AN treatment (*n* = 16 per groups). **E** Representative BODIPY-ATP fluorescence images in D2 embryos with and without AN treatment. Scale bar = 50 μm. **F** Relative ATP fluorescence intensity in D2 embryos with and without AN treatment (*n* = 24 per groups). Data are from three independent experiments, and different superscript letters indicate a significant difference (*P* < 0.05)

We investigated the lipid droplet, fatty acid, and ATP contents in D2 embryos. The levels of lipid droplets and fatty acids were significantly higher in D2 embryos supplemented with AN than in the control (Fig. 4A–D). The ATP level was significantly increased by AN compared to the control (Fig. 4E and F).

### AN modulates SHH signaling during porcine IVF embryo development

**Fig. 5:**
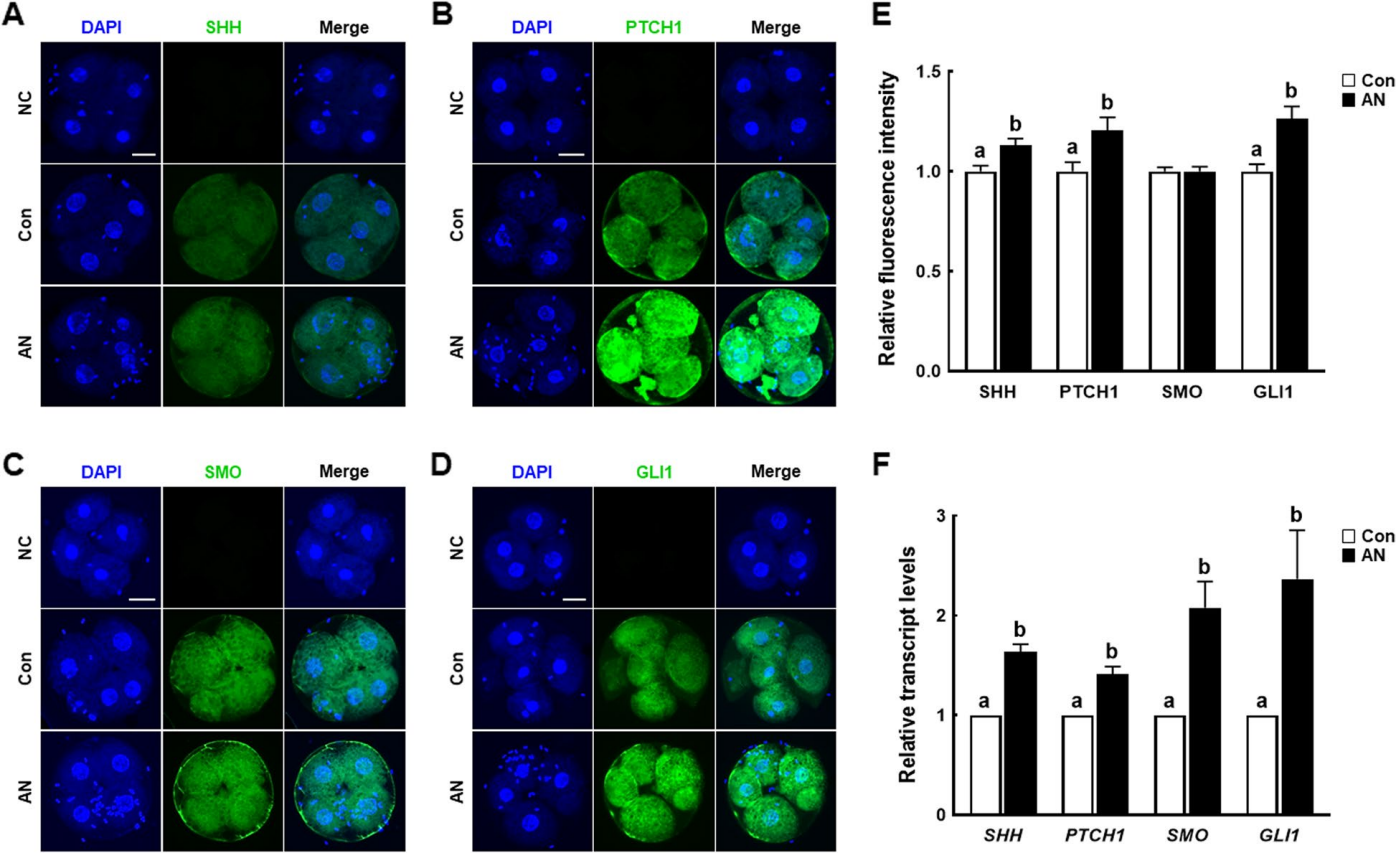
Effect of AN on the levels of proteins related to sonic hedgehog (SHH) signaling in porcine IVF embryos. Representative immunofluorescence images of **A** SHH, **B** PTCH1, **C** SMO, and **D** GLI1 in D2 embryos with and without AN treatment. Scale bar = 50 μm. **E** Relative fluorescence intensities of SHH, PTCH1, SMO, and GLI1 in D2 embryos with and without AN treatment (*n* = 30 per groups). **F** Relative expression levels of SHH signaling-related genes in D2 embryos with and without AN treatment (*n* = 3 per groups). Data are from three independent experiments, and different superscript letters indicate a significant difference (*P* < 0.05)

We examined the level of SHH signaling pathway-related proteins (SHH, SMO, PTCH1, and GLI1) in D2 embryos. The levels of SHH, PTCH1, and GLI1 were significantly increased in the AN group compared to the control. However, no difference in SMO immunostaining was observed in the AN group (Fig. 5A–E). The expression levels of SHH signaling pathway-related genes were significantly higher in D2 embryos in the AN group (Fig. 5F). To further validate that SHH signaling pathway is related to the mechanism by which AN improves developmental competences of porcine IVF embryo, we cultured IVF embryos treated with AN with or without cyclopamine and assessed the cleavage and blastocyst rates on 2 and 6 d, respectively. AN-mediated improvements in the developmental competences were significantly reduced by cotreatment with cyclopamine (Additional files 6, 7, 8, 9, 10: Table S6–9, Fig. S1).

## Discussion

**Fig. 6:**
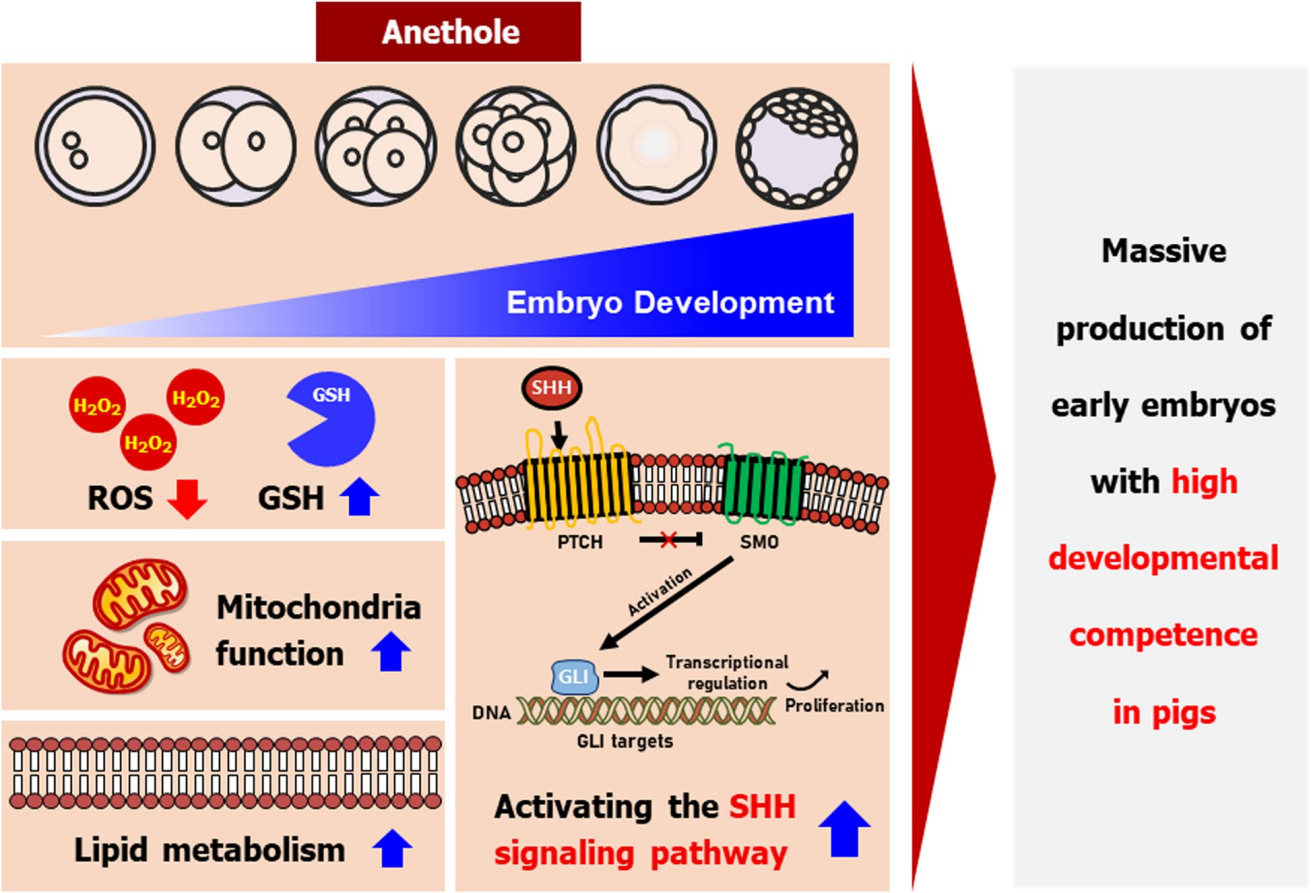
Graphical overview of AN effects on porcine embryonic development. AN improved the preimplantation embryonic development on porcine. In particular, AN improves the mitochondria function and lipid metabolism, and activates SHH signaling pathway. These finding suggest that AN improves the developmental competence of porcine embryo by activating the SHH signaling against oxidative stress

During IVC, embryos are sensitive to oxidative stress due to differences between the in vivo environment and IVC systems. ROS-induced oxidative stress causes a variety of impairments in developmental parameters (e.g., blastocyst formation rate, TE cell number, and cellular survival) [31]. Thus, supplementation with antioxidants to prevent excessive ROS accumulation can improve embryonic developmental competence. We investigated the effect of AN on porcine preimplantation embryos. AN supplementation during IVC markedly improved developmental competence by reducing intracellular ROS production, enhancing mitochondrial function, and regulating lipid metabolism. Moreover, AN supplementation significantly increased the expression of SHH signaling-related proteins and genes. These results suggest that AN enhances preimplantation embryo development by regulating oxidative stress and activating the SHH signaling pathway (Fig. 6).

AN is present at a high concentration in fennel oil, which has antioxidant properties. AN ameliorated colitis in the colon tissue via anti-inflammatory and antioxidant effects [32]. Furthermore, AN exerted anti-inflammatory, antioxidant, and anti-apoptotic effects in renal ischemia/reperfusion-induced injury and exhibited renoprotective activity by inhibiting the HMGB1/TLR2, 4/MYD88/NF-κB pathway [33]. AN supplementation improved the development of preantral follicles and the proportion of oocytes with the ability to resume meiosis by decreasing the ROS level in goat [20]. Consistent with previous studies, AN supplementation improved the developmental competence of porcine IVF embryos in terms of the blastocyst formation rate, cell number, and cellular survival rate. The reason why there was no difference in cleavage rate might be due to the fact that high rates (more than 80%) of cleavage rates are already achieved with our IVC system [8, 34, 35]. Moreover, AN supplementation reduced ROS levels and increased the GSH level and the expression levels of antioxidant genes. These results suggest that AN promotes porcine embryonic development by reducing oxidative stress.

TE is the first cell type to appear during mammalian embryogenesis and is crucial for viviparous reproduction in placental mammals. TE, with its typical epithelial morphology, surrounds a fluid-filled cavity whose expansion is critical for hatching and efficient interaction with the uterine endometrium for implantation [36]. TE differentiates into trophoblast cells to construct the placenta, and the number or proportion of TE cells is frequently associated with the degree of cellular apoptosis, which influences implantation efficiency [37]. The fate of an implanted embryo can be predicted based on TE quality and cell number at the blastocyst stage; in this way, live birth and early pregnancy losses can be distinguished [38]. Thus, TE development during preimplantation stages is a necessary antecedent to the events of implantation [39]. In this study, AN supplementation during IVC significantly increased the TE cell number and *CDX2* expression, indicating that AN enhances proliferation or differentiation of TE cells.

Mitochondria are essential organelles in embryos for cellular metabolism and function. Excessive ROS production negatively affects mitochondria, impairing embryonic developmental competency and inducing cell cycle arrest and apoptosis [25, 40]. Moreover, oxidative stress decreases the cellular mitochondria content by disturbing mitochondrial biogenesis and function [41]. The mitochondrial membrane potential is an indicator of mitochondrial function. Embryos with a high mitochondria membrane potential exert positive effects on developmental potential of porcine preimplantation embryos [42]. In this study, AN supplementation improved mitochondrial function by increasing mitochondrial content and mitochondrial membrane potential in porcine IVF embryos. This is consistent with a prior report that AN increased the mitochondrial membrane potential and antioxidant activity in bovine oocytes [21]. Therefore, AN exerts a beneficial effect on porcine preimplantation embryos by increasing mitochondrial function.

Lipid metabolism is important in embryonic development as a source of energy [43]. Lipid droplets are crucial storage organelles at the center of lipid and energy homeostasis, especially in species with lipid-rich oocytes, providing indispensable substrates for early embryonic development before zygotic genome activation [44]. Lipid droplets are synthesized using fatty acids derived from liquid–liquid phase separation and lipids accumulated in the membrane of the endoplasmic reticulum and reduced the size through triglyceride form by lipase activation. The released fatty acids are transported to mitochondria and undergo β-oxidation for ATP production [44–46]. However, oxidative stress exerts a detrimental effect on embryonic development by reducing the ATP level and lipid peroxidation [47]. In this study, AN supplementation significantly enhanced lipid metabolism by increasing the levels of lipid droplets and fatty acids, enhancing lipid metabolism-derived ATP production. These results are consistent with a report that supplementation of pigs with dietary dihydromyricetin, which is an antioxidant, improved lipid metabolism [48]. In short, AN supplementation during IVC enhanced lipid metabolism and ATP production in porcine embryos.

The SHH signaling pathway is implicated in vertebrate embryonic development and in cell proliferation and differentiation via paracrine signaling. Genes and proteins related to the SHH signaling pathway are expressed at various stages of development in porcine parthenogenetic embryos [49]. SHH supplementation during IVC significantly improved the developmental competence of porcine and goat IVF embryos [29, 50]. In addition, mice deficient in the *SHH* gene have holoprosencephaly (smaller head) with cyclopia (single eye), and lack ventral cell types within the neural tube of the spinal cord and in most of the ribs; *SHH*-knockout mice die by 10.5 d [51]. Previous studies reported that exogenous recombinant SHH increased the activities of anti-oxidant enzymes and prevented apoptosis through regulating pro-apoptotic/anti-apoptotic genes and overactivation of ERK under oxidative stress condition; in particular, these protective effects were partially reversed by cyclopamine [52–54]. In this study, AN supplementation significantly increased the levels of SHH signaling pathway-related proteins (SHH, PTCH1, and GLI1), possibly promoting preimplantation embryo development and reducing ROS generation. These results are consistent with a report that baicalin treatment during IVC improved in vitro development of porcine parthenogenetic and IVF embryos with active SHH signaling [25]. However, AN did not significantly influence SMO expression, possibly because this gene is regulated post-transcriptionally, whereas PTCH1 and GLI1 are regulated transcriptionally [55]. Therefore, AN supplementation during IVC enhances the developmental competence of porcine IVF embryos by activating the SHH signaling pathway.

## Conclusions

This is the first study of the effect of AN on porcine embryonic development. AN supplementation during IVC reduced oxidative stress and increased mitochondrial function and lipid metabolism by activating the SHH signaling pathway, enhancing the developmental competence of porcine IVF embryos. These findings suggest AN to be useful for large-scale production of high-quality porcine embryos and potentially for the treatment of human infertility.

## Supplementary Information

**Additional file 1: Table S1.** Primer sequences for qRT-PCR. 

**Additional file 2: Table S2.** Effect of anethole (AN) concentrations on in vitro development of porcine in vitro fertilization (IVF) embryos.

**Additional file 3: Table S3.** Effects of AN on the post-blastulation development of porcine IVF blastocysts.

**Additional file 4: Table S4.** Effects of AN on cell survival in porcine IVF blastocysts. 

**Additional file 5: Table S5.** Effects of AN on inner cell mass (ICM), trophectoderm (TE) and total cell number in porcine IVF blastocysts.

**Additional file 6: Table S6.** Effect of AN with or without cyclopamine on in vitro development of porcine IVF embryos.

**Additional file 7: Table S7.** Effects of AN with or without cyclopamine on the post-blastulation development of porcine IVF blastocysts.

**Additional file 8: Table S8.** Effects of AN with or without cyclopamine on cell survival in porcine IVF blastocysts.

**Additional file 9: Table S9.** Effects of AN with or without cyclopamine on ICM, TE and total cell number in porcine IVF blastocysts.

**Additional file 10: Fig. S1.** Effect of AN with or without cyclopamine on the development of porcine IVF embryos. (**A**) Representative images of embryos on D6, (**B**) percentages of cleavage on D2, and (**C**) blastocyst formation on D6 (*n* = 175 per groups). Scale bar = 100 μm. (**D**) Proportions of blastocyst stages after treatment of AN with or without cyclopamine treatment (Con; *n* = 53, AN; n = 78, AN+Cy; *n* = 52). (**E**) Representative photographs of terminal deoxynucleotidyl transferase-mediated dUTP-digoxygenin staining of blastocysts on D6 and (**F**) percentages of apoptotic cells after treatment of AN with or without cyclopamine treatment (*n* = 20 per groups). Scale bar = 50 μm. (**G**) Representative images of CDX2 staining of D6 blastocysts and (**H**) numbers of ICM, TE, and total cells in D6 blastocysts after treatment of AN with or without cyclopamine treatment (*n* = 22 per groups). Scale bar = 50 μm. Data are from five independent experiments, and different superscript letters indicate a significant difference (*P* < 0.05).

## Data Availability

All data generated or analyzed during this study are included in this published article.

## Abbreviations

AN: Anethole
ARTs: Assisted reproductive technologies
ATP: Adenosine 5'-triphosphate
BSA: Bovine serum albumin
CAT: Catalase
COCs: Cumulus oocyte complexes
DPBS: Dulbecco’s phosphate-buffered saline
ExB: Expanded blastocysts
GLI: Glioma-associated oncogene homolog
GPX: Glutathione peroxidase
GSH: Glutathione
ICM: Inner cell mass
IVC: In vitro culture
IVF: In vitro fertilization
IVM: In vitro maturation
PTCH: Patched
PVA: Polyvinyl alcohol
ROS: Reactive oxygen species
RT: Room temperature
SHH: Sonic hedgehog
SMO: Smoothened
SOD: Superoxide dismutase
TE: Trophectoderm
