# Supplementary material for: Anethole improves the developmental competence of porcine embryos by reducing oxidative stress via the sonic hedgehog signaling pathway
Source: J Anim Sci Biotechnol. 2023 Feb 22;14:32. doi: 10.1186/s40104-022-00824-x (PMC9945695; doi:10.1186/s40104-022-00824-x)
Supplement: Supplementary file 1 — Additional file 1: Table S1. Primer sequences for qRT-PCR. [file 40104_2022_824_MOESM1_ESM.docx]

**Table S1** Primer sequences for qRT-PCR

| **Gene** | **Primer sequences (5’→3’)** | **Gene accession no.** | **Length, bp** |
| --- | --- | --- | --- |
| ***H2A*** | F: AGTTTCCTGTGGGTCGAGTG | XM_021083382.1 | 162 |
|  | R: TGCGAGTCTTCTTGTTGTC |  |  |
| ***OCT4*** | F: AGTGAGAGGCAACCTGGAGA | NM_001113060.1 | 151 |
|  | R: ACTGCTTGATCGTTTGCCCT |  |  |
| ***CDX2*** | F: GGCAGCCAAGTGAAAACCAG | XM_003127290.5 | 251 |
|  | R: GCCTTTCTCCGAATGGTGAT |  |  |
| ***BCL-XL*** | F: AGGGCATTCAGTGACCTGAC | NM_214285 | 242 |
|  | R: TGGATCCAAGGCTCTAGGTG |  |  |
| ***BAX*** | F: AAGCGCATTGGAGATGAACT | XM_003127290 | 251 |
|  | R: CGATCTCGAAGGAAGTCCAG |  |  |
| ***SOD1*** | F: GGTGGGCCAAAGGATCAAGA | NM_001190422.1 | 80 |
|  | R: TACACAGTGGCCACACCATC |  |  |
| ***SOD2*** | F: GGTGGAGGCCACATCAATCA | NM_214127.2 | 220 |
|  | R: AACAAGCGGCAATCTGCAAG |  |  |
| ***CAT*** | F: TGTACCCGCTATTCTGGGGA | NM_214301.2 | 119 |
|  | R: TCACACAGGCGTTTCCTCTC |  |  |
| ***GPX1*** | F: TGGACATCAGGAAAATGCCAAG | NM_214201.1 | 127 |
|  | R: GTGAGCATTTGCGCCATTCA |  |  |
