# Supplementary material for: Anethole improves the developmental competence of porcine embryos by reducing oxidative stress via the sonic hedgehog signaling pathway
Source: J Anim Sci Biotechnol. 2023 Feb 22;14:32. doi: 10.1186/s40104-022-00824-x (PMC9945695; doi:10.1186/s40104-022-00824-x)
Supplement: Supplementary file 2 — Additional file 2: Table S2. Effect of anethole (AN) concentrations on in vitro development of porcine in vitro fertilization (IVF) embryos. [file 40104_2022_824_MOESM2_ESM.docx]

Table S2 Effect of anethole (AN) concentrations on *in vitro* development of porcine in vitro fertilization (IVF) embryos

| **Anethole, mg/mL** | **No. of embryos examined** | **Cleavage, %** | **Blastocyst, %** |
| --- | --- | --- | --- |
| 0 | 132 | 108 (81.7±5.4) | 30 (22.8±2.2)^a^ |
| 0.3 | 134 | 117 (86.7±5.1) | 40 (30.6±3.2)^ab^ |
| 0.5 | 134 | 111 (82.0±3.6) | 50 (38.2±3.0)^b^ |
| 1 | 133 | 116 (86.7±2.4) | 30 (23.0±1.9)^a^ |

Data are the mean ± SEM, and values with different superscript letter within a column differ significantly (*P* < 0.05)
