# Supplementary material for: Anethole improves the developmental competence of porcine embryos by reducing oxidative stress via the sonic hedgehog signaling pathway
Source: J Anim Sci Biotechnol. 2023 Feb 22;14:32. doi: 10.1186/s40104-022-00824-x (PMC9945695; doi:10.1186/s40104-022-00824-x)
Supplement: Supplementary file 3 — Additional file 3: Table S3. Effects of AN on the post-blastulation development of porcine IVF blastocysts. [file 40104_2022_824_MOESM3_ESM.docx]

Table S3 Effects of AN on the post-blastulation development of porcine IVF blastocysts

| **Groups** | **No. of**  **blastocysts examined** | **Proportion of blastocysts developed to the following stages, %** | | |
| --- | --- | --- | --- | --- |
|  |  | **Early** | **Middle** | **Expanded** |
| Con | 40 | 16.0±2.1 | 59.0±4.9^a^ | 25.7±6.2^a^ |
| AN | 40 | 12.3±3.3 | 23.3±3.3^b^ | 53.3±3.3^b^ |

Data are the mean ± SEM, and values with different superscript letter within a column differ significantly (*P* < 0.05)
