# Supplementary material for: Anethole improves the developmental competence of porcine embryos by reducing oxidative stress via the sonic hedgehog signaling pathway
Source: J Anim Sci Biotechnol. 2023 Feb 22;14:32. doi: 10.1186/s40104-022-00824-x (PMC9945695; doi:10.1186/s40104-022-00824-x)
Supplement: Supplementary file 4 — Additional file 4: Table S4. Effects of AN on cell survival in porcine IVF blastocysts. [file 40104_2022_824_MOESM4_ESM.docx]

Table S4 Effects of AN on cell survival in porcine IVF blastocysts

| **Groups** | **No. of blastocysts examined** | **No. of TUNEL-positive cells** | **Apoptosis, %** |
| --- | --- | --- | --- |
| Con | 35 | 1.6±0.2 | 4.9±0.7^a^ |
| AN | 35 | 1.4±0.2 | 3.2±0.5^b^ |

Data are the mean ± SEM, and values with different superscript letter within a column differ significantly (*P* < 0.05)
