# Supplementary material for: Anethole improves the developmental competence of porcine embryos by reducing oxidative stress via the sonic hedgehog signaling pathway
Source: J Anim Sci Biotechnol. 2023 Feb 22;14:32. doi: 10.1186/s40104-022-00824-x (PMC9945695; doi:10.1186/s40104-022-00824-x)
Supplement: Supplementary file 5 — Additional file 5: Table S5. Effects of AN on inner cell mass (ICM), trophectoderm (TE) and total cell number in porcine IVF blastocysts. [file 40104_2022_824_MOESM5_ESM.docx]

Table S5 Effects of AN on inner cell mass (ICM), trophectoderm (TE) and total cell number in porcine IVF blastocysts

| **Groups** | **No. of blastocysts examined** | **No. of nuclei** | | |
| --- | --- | --- | --- | --- |
|  |  | **ICM** | **TE** | **Total** |
| Con | 26 | 9.5±0.6 | 23.1±1.0^a^ | 32.7±1.2^a^ |
| AN | 26 | 10.9±0.9 | 30.7±1.6^b^ | 41.6±1.9^b^ |

Data are the mean ± SEM, and values with different superscript letter within a column differ significantly (*P* < 0.05)
