# Supplementary material for: Anethole improves the developmental competence of porcine embryos by reducing oxidative stress via the sonic hedgehog signaling pathway
Source: J Anim Sci Biotechnol. 2023 Feb 22;14:32. doi: 10.1186/s40104-022-00824-x (PMC9945695; doi:10.1186/s40104-022-00824-x)
Supplement: Supplementary file 6 — Additional file 6: Table S6. Effect of AN with or without cyclopamine on in vitro development of porcine IVF embryos. [file 40104_2022_824_MOESM6_ESM.docx]

Table S6 Effect of AN with or without cyclopamine on in vitro development of porcine IVF embryos

| **Groups** | **No. of embryos examined** | **Cleavage, %** | **Blastocyst, %** |
| --- | --- | --- | --- |
| Control | 175 | 140 (80.0±3.5) | 53 (30.3±2.9)^a^ |
| Anethole | 175 | 146 (83.4±1.1) | 78 (44.6±2.5)^b^ |
| Anethole+Cyclopamine | 175 | 134 (76.6±4.7) | 52 (29.7±3.1)^a^ |

Data are the mean ± SEM, and values with different superscript letter within a column differ significantly (*P* < 0.05)
