# Supplementary material for: Anethole improves the developmental competence of porcine embryos by reducing oxidative stress via the sonic hedgehog signaling pathway
Source: J Anim Sci Biotechnol. 2023 Feb 22;14:32. doi: 10.1186/s40104-022-00824-x (PMC9945695; doi:10.1186/s40104-022-00824-x)
Supplement: Supplementary file 7 — Additional file 7: Table S7. Effects of AN with or without cyclopamine on the post-blastulation development of porcine IVF blastocysts. [file 40104_2022_824_MOESM7_ESM.docx]

Table S7 Effects of AN with or without cyclopamine on the post-blastulation development of porcine IVF blastocysts

| **Groups** | **No. of**  **blastocysts examined** | **Proportion of blastocysts developed to the following stages, %** | | |
| --- | --- | --- | --- | --- |
|  |  | **Early** | **Middle** | **Expanded** |
| Con | 53 | 16.5±2.2 | 48.2±3.1^a^ | 35.4±2.0^a^ |
| AN | 78 | 15.4±1.4 | 33.0±4.3^b^ | 51.6±4.1^b^ |
| AN+Cy | 52 | 17.4±1.4 | 57.0±3.3^a^ | 25.6±2.5^a^ |

Data are the mean ± SEM, and values with different superscript letter within a column differ significantly (*P* < 0.05)
