# Supplementary material for: Anethole improves the developmental competence of porcine embryos by reducing oxidative stress via the sonic hedgehog signaling pathway
Source: J Anim Sci Biotechnol. 2023 Feb 22;14:32. doi: 10.1186/s40104-022-00824-x (PMC9945695; doi:10.1186/s40104-022-00824-x)
Supplement: Supplementary file 8 — Additional file 8: Table S8. Effects of AN with or without cyclopamine on cell survival in porcine IVF blastocysts. [file 40104_2022_824_MOESM8_ESM.docx]

Table S8 Effects of AN with or without cyclopamine on cell survival in porcine IVF blastocysts

| **Groups** | **No. of blastocysts examined** | **No. of TUNEL-positive cells** | **Apoptosis, %** |
| --- | --- | --- | --- |
| Con | 20 | 2.3±0.3^a,b^ | 5.3±0.7^a^ |
| AN | 20 | 1.5±0.2^b^ | 2.8±0.3^b^ |
| AN+Cy | 20 | 3.1±0.4^a^ | 7.0±0.8^a^ |

Data are the mean ± SEM, and values with different superscript letter within a column differ significantly (*P* < 0.05)
