# Supplementary material for: Anethole improves the developmental competence of porcine embryos by reducing oxidative stress via the sonic hedgehog signaling pathway
Source: J Anim Sci Biotechnol. 2023 Feb 22;14:32. doi: 10.1186/s40104-022-00824-x (PMC9945695; doi:10.1186/s40104-022-00824-x)
Supplement: Supplementary file 9 — Additional file 9: Table S9. Effects of AN with or without cyclopamine on ICM, TE and total cell number in porcine IVF blastocysts. [file 40104_2022_824_MOESM9_ESM.docx]

Table S9 Effects of AN with or without cyclopamine on ICM, TE and total cell number in porcine IVF blastocysts

| **Groups** | **No. of blastocysts examined** | **No. of nuclei** | | |
| --- | --- | --- | --- | --- |
|  |  | **ICM** | **TE** | **Total** |
| Con | 22 | 7.5±1.1 | 30.9±2.1^a^ | 38.4±2.3^a^ |
| AN | 22 | 7.5±0.7 | 48.8±2.9^b^ | 56.3±2.9^b^ |
| AN+Cy | 22 | 7.0±0.8 | 30.2±1.3^a^ | 37.2±1.3^a^ |

Data are the mean ± SEM, and values with different superscript letter within a column differ significantly (*P* < 0.05)
