# Supplementary figures and images for: Anethole improves the developmental competence of porcine embryos by reducing oxidative stress via the sonic hedgehog signaling pathway
Source: J Anim Sci Biotechnol. 2023 Feb 22;14:32. doi: 10.1186/s40104-022-00824-x (PMC9945695; doi:10.1186/s40104-022-00824-x)

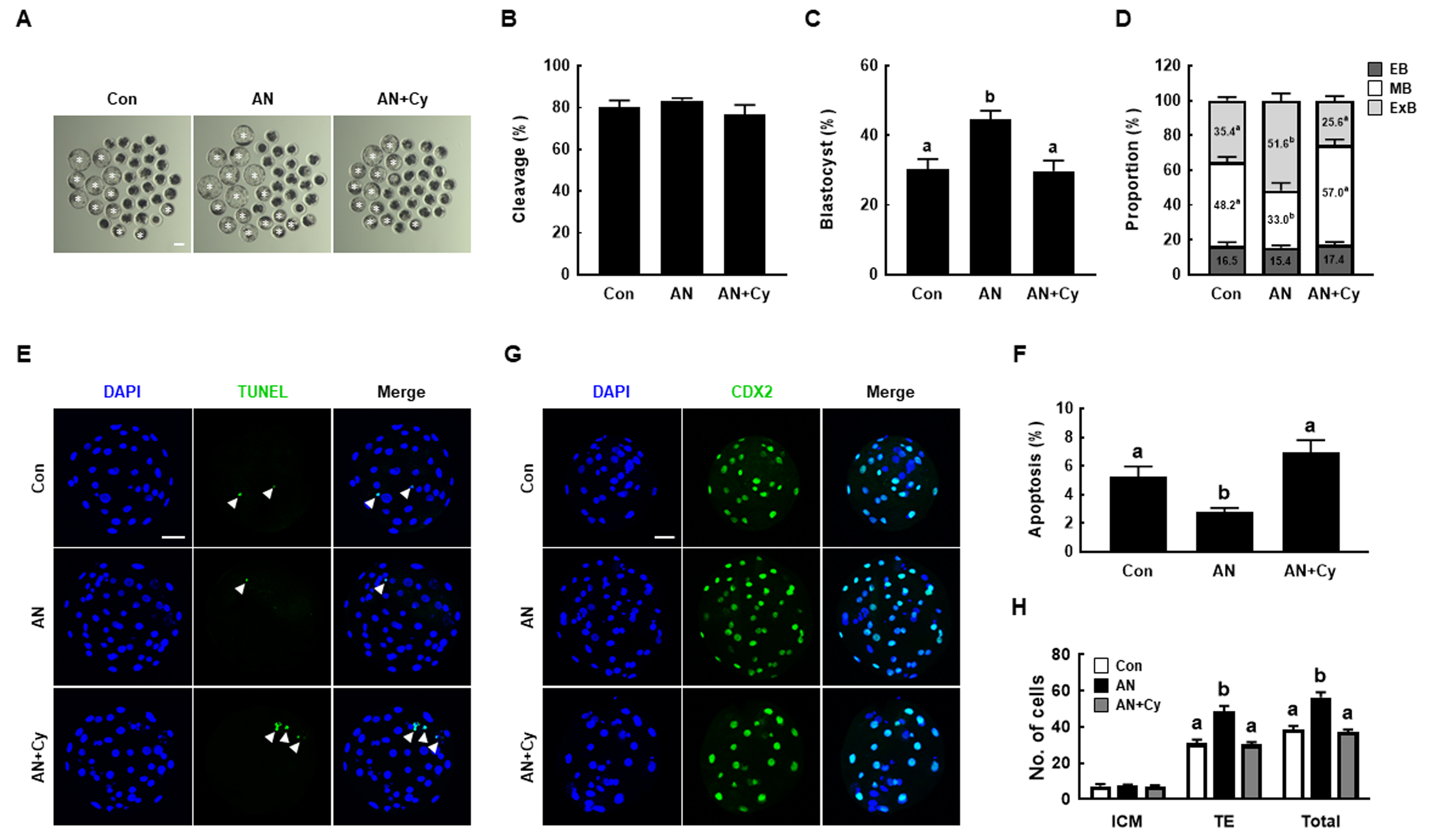

Supplement: Supplementary file 10 — Additional file 10: Fig. S1. Effect of AN with or without cyclopamine on the development of porcine IVF embryos. (A) Representative images of embryos on D6, (B) percentages of cleavage on D2, and (C) blastocyst formation on D6 (n = 175 per groups). Scale bar = 100 μm. (D) Proportions of blastocyst stages after treatment of AN with or without cyclopamine treatment (Con; n = 53, AN; n = 78, AN+Cy; n = 52). (E) Representative photographs of terminal deoxynucleotidyl transferase-mediated dUTP-digoxygenin staining of blastocysts on D6 and (F) percentages of apoptotic cells after treatment of AN with or without cyclopamine treatment (n = 20 per groups). Scale bar = 50 μm. (G) Representative images of CDX2 staining of D6 blastocysts and (H) numbers of ICM, TE, and total cells in D6 blastocysts after treatment of AN with or without cyclopamine treatment (n = 22 per groups). Scale bar = 50 μm. Data are from five independent experiments, and different superscript letters indicate a significant difference (P < 0.05). [file 40104_2022_824_MOESM10_ESM.tif]
